# Hedgehog pathway inhibitors for locally advanced and metastatic basal cell carcinoma: A real-world single-center retrospective review

**DOI:** 10.1371/journal.pone.0297531

**Published:** 2024-04-30

**Authors:** Shivani Patel, Heather Armbruster, Gretchen Pardo, Brianna Archambeau, Na Hyun Kim, Joanne Jeter, Richard Wu, Kari Kendra, Carlo M. Contreras, Natalie Spaccarelli, Brittany Dulmage, Llana Pootrakul, David R. Carr, Claire Verschraegen

**Affiliations:** 1 Department of Pharmacy, The James Cancer Hospital, Columbus, OH, United States of America; 2 Celltrion, Incheon, South Korea; 3 Huntsman Cancer Institute, University of Utah, Salt Lake City, UT, United States of America; 4 Division of Medical Oncology, The Ohio State University Comprehensive Cancer Center, Columbus, OH, United States of America; 5 Department of Surgery, The Ohio State University Wexner Medical Center, Columbus, OH, Unites States of America; 6 Department of Dermatology, The Ohio State University Wexner Medical Center, Columbus, OH, Unites States of America; Maria Sklodowska-Curie National Research Institute of Oncology Krakow, POLAND

## Abstract

Basal cell carcinoma (BCC) is highly curable by surgical excision or radiation. In rare cases, BCC can be locally destructive or difficult to surgically remove. Hedgehog inhibition (HHI) with vismodegib or sonidegib induces a 50–60% response rate. Long-term toxicity includes muscle spasms and weight loss leading to dose decreases. This retrospective chart review also investigates the impact of CoQ10 and calcium supplementation in patients treated with HHI drugs at a single academic medical center from 2012 to 2022. We reviewed the charts of adult patients diagnosed with locally advanced or metastatic BCC treated with vismodegib or sonidegib primarily for progression-free survival (PFS). Secondary objectives included overall survival, BCC-specific survival, time to and reasons for discontinuation, overall response rate, safety and tolerability, use of CoQ10 and calcium supplements, and insurance coverage. Of 55 patients assessable for outcome, 34 (61.8%) had an overall clinical benefit, with 25 (45.4%) having a complete response and 9 (16.3%) a partial response. Stable disease was seen in 14 (25.4%) and 7 (12.7%) progressed. Of the 34 patients who responded to treatment, 9 recurred. Patients who were rechallenged with HHI could respond again. The median overall BCC-specific survival rate at 5 years is 89%. Dose reductions or discontinuations for vismodegib and sonidegib occurred in 59% versus 24% of cases, or 30% versus 9% of cases, respectively. With CoQ10 and calcium supplementation, only 17% required a dose reduction versus 42% without. HHI is highly effective for treating advanced BCC but may require dosing decreases. Sonidegib was better tolerated than vismodegib. CoQ10 and calcium supplementation can effectively prevent muscle spasms.

## 1. Background

Basal cell carcinoma (BCC) is the most common skin cancer in people of Caucasian ancestry, affecting 5.4 million people worldwide each year [1]. Major risk factors for BCC include: prolonged exposure to UV light, especially of the UVB wavelength [2]; genetic predisposition; lack of pigmentation; ionizing radiation exposure; immunosuppression; some genetic syndromes, the most common one being basal cell nevus syndrome (BCNS, or Gorlin syndrome) [1, 3, 4]; or exposure to toxins, such as arsenic. BCC is usually a slow-growing tumor with good prognosis that rarely metastasizes. Most patients are cured after surgical resection or ablation. However, if neglected, BCC can lead to significant local tissue destruction and disfigurement (locally advanced BCC (laBCC)) and rarely distant metastases (metastatic BCC (mBCC)) requiring systemic therapy [5]. The standard-of-care first-line systemic treatment consists of US Food and Drug Administration (FDA)-approved hedgehog inhibitors (HHIs) [6] (either vismodegib (Erivedge®) [7] or sonidegib (Odomzo®) [8]). Second-line therapy is immunotherapy with cemiplimab, a monoclonal antibody PD-1 inhibitor, approved in 2021 for patients who have disease progression while on HHIs or for whom HHIs are not appropriate [9].

The most common adverse event experienced with HHI treatment is muscle spasm, affecting nearly half of patients. In studies of embryogenesis, a gradient of Sonic hedgehog (SHH) is necessary for normal neurological development. SHH binds to its receptor, Patched (PTCH), which recruits and activates Smoothened (SMO) to regulate gene expression [10]. BCC oncogenesis has not been well studied, but is linked to mutations involving mainly the Patched-1 (PTCH1) [11] and SMO genes [12, 13]. These activating mutations lead to stimulation of the hedgehog pathway and its targets, the GLI1 and GLI2 transcription factors. SMO is a 7-transmembrane receptor that activates G protein-coupled receptors that often trigger second messengers such as Ca^2+^. Thus, SHH acutely increases Ca^2+^ spike activity through activation of the SHH coreceptor SMO, as demonstrated in neurons, causing both intracellular Ca^2+^ stores and Ca^2+^ influx. Therefore, inhibition of SHH depletes the muscle cells of Ca^2+^, inducing muscle spasms. Consequently, calcium replacement might prevent muscle spasms in patients taking HHIs. Similarly, there are some studies showing a trend for coenzyme Q10 (CoQ10) to help support muscle health [14–16], so ubiquinone supplementation together with calcium supplementation was of interest in lowering the risk of muscle cramping while on HHIs.

The main objectives of this retrospective study of patients with laBCC and mBCC treated at a single academic center over the last 10 years were to evaluate the efficacy of HHIs and focus on the mitigation of side effects. This review will provide a benchmark for real-world patients affected by advanced BCCs treated with this class of oral targeted therapy and assess the potential impact of calcium and CoQ10 supplementation in patients undergoing HHI therapy.

## 2. Methods

This is a single-center retrospective review evaluating the efficacy and safety of HHIs in patients with locally advanced and metastatic BCC receiving standard-of-care treatments. Written institutional review board (IRB) approval was obtained before starting the chart reviews, and a waiver of consent was granted. Data was collected between December 2021 and August 2022. Only the first and corresponding authors had access to patient data during data collection. After data was collected and curated, it was anonymized. Data was accessed for research purposes intermittently from August 2022 until February 2023.

### 2.1 Endpoints

The primary objective was to assess progression-free survival (PFS). PFS was measured from the start of HHI treatment to evidence of targeted BCC recurrence, start of a different class of BCC therapy, death, or lost to follow-up.

The secondary objectives included the assessment of overall survival (OS); the safety and tolerability of HHIs, including reported adverse events and their grade; and the use of concomitant CoQ10, calcium supplements, and/or other medications to prevent the musculoskeletal toxicity related to HHIs. OS was measured from start of treatment to the date of death or last date known to be alive per medical records review. Both BCC-specific and other causes of mortality were recorded.

Ancillary objectives included: response to HHI; dosage adjustments; reasons for dosage adjustments; rates of interruption and discontinuation; determination of a maintenance dose if any; crossover from vismodegib to sonidegib and vice versa; subsequent line of treatments in patients who discontinued HHI; and determination of accessibility of HHI, reviewing insurance approval, copay needs, and access to manufacturer (Novartis/Genentech) or non-manufacturer program assistance (for example, grant support). Responses were extracted from patient electronic medical records (EMRs) via clinic notes, pathology biopsies, pictures in the electronic medical records, and imaging. Given the retrospective nature of this study, it was not possible to assess the time to response from the notes, thus only the response type, PFS, and OS were collected.

### 2.2 Patient eligibility

Eligible patients had a locally advanced or metastatic BCC diagnosis confirmed through a pathology report, were age 18 years and older, and received an HHI for their condition prescribed by an Ohio State University Comprehensive Cancer Center (OSUCCC) medical oncologist with a documented start of treatment in the EMR. Pregnant patients, prisoners, patients enrolled in an HHI clinical trial, and patients taking an HHI prescribed by a provider at another institution were excluded.

### 2.3 Data collection

Patients were identified by treatment plans for vismodegib or sonidegib with ICD-10 codes for BCC, laBCC, or mBCC. Data collected from the EMRs included demographics (age, gender, Fitzpatrick Phototype Classification); weight; HHI drug name; initial regimen and changes in regimen; treatment start and stop dates; and adverse events, especially musculoskeletal issues, dysgeusia, and any other likely related side effects. Grading was inferred from the side effect descriptions and based on the National Cancer Institute (NCI) Common Terminology Criteria for Adverse Events v5.0 [17]. Concomitant use of CoQ10 and calcium supplements was recorded. Reason and date of treatment interruption, resumption, or discontinuation and whether a maintenance regimen was established were recorded. A maintenance regimen was defined as a treatment regimen continued for more than 6 months to maintain steady response in the absence of disease progression as documented in the progress notes in the EMR. Crossover between two HHIs or a subsequent line of treatment and the reason why were recorded. Both BCC-specific and other causes of mortality were recorded. Response definition is per RECIST criteria [18]. Responses were assessed at 4 months (± 1 month).

The survey period began January 30, 2012 and ended June 30, 2021, with a follow-up period of 6 months for response and adverse events after HHI discontinuation. An updated analysis of patients’ overall survival was then done up to July 31, 2022.

### 2.4 Statistical analyses

Descriptive statistics were used to analyze baseline characteristics and safety outcomes. For continuous variables, median and range were provided. Initial treatment regimen and changes in regimen, adverse events, calcium and/or CoQ10 supplementation, maintenance regimen, subsequent treatments, and accessibility were presented using descriptive statistics with frequency and percentage for categorical variables. The time scale is presented in months and there are no adjustment factors. In this PFS analysis, we used real-world PFS to account for real-world practice [19]. The log-rank test was used to calculate the p-value for the comparison between receiving sonidegib and vismodegib, with an alpha level of 0.05. Estimates and corresponding 95% confidence intervals (CIs) were based on the Kaplan-Meier method to evaluate progression-free survival, overall survival, time to treatment discontinuation, time to treatment interruption, and time to treatment failure. With these retrospective data, exploratory analysis was conducted with various methods (non-parametric method and semi-parametric method). Statistical Analysis System (SAS^®^) software, version 9.4 (SAS Institute Inc., Cary, NC, USA) was used for statistical analysis.

## 3. Results

### 3.1 Patient characteristics

**Fig 1:**
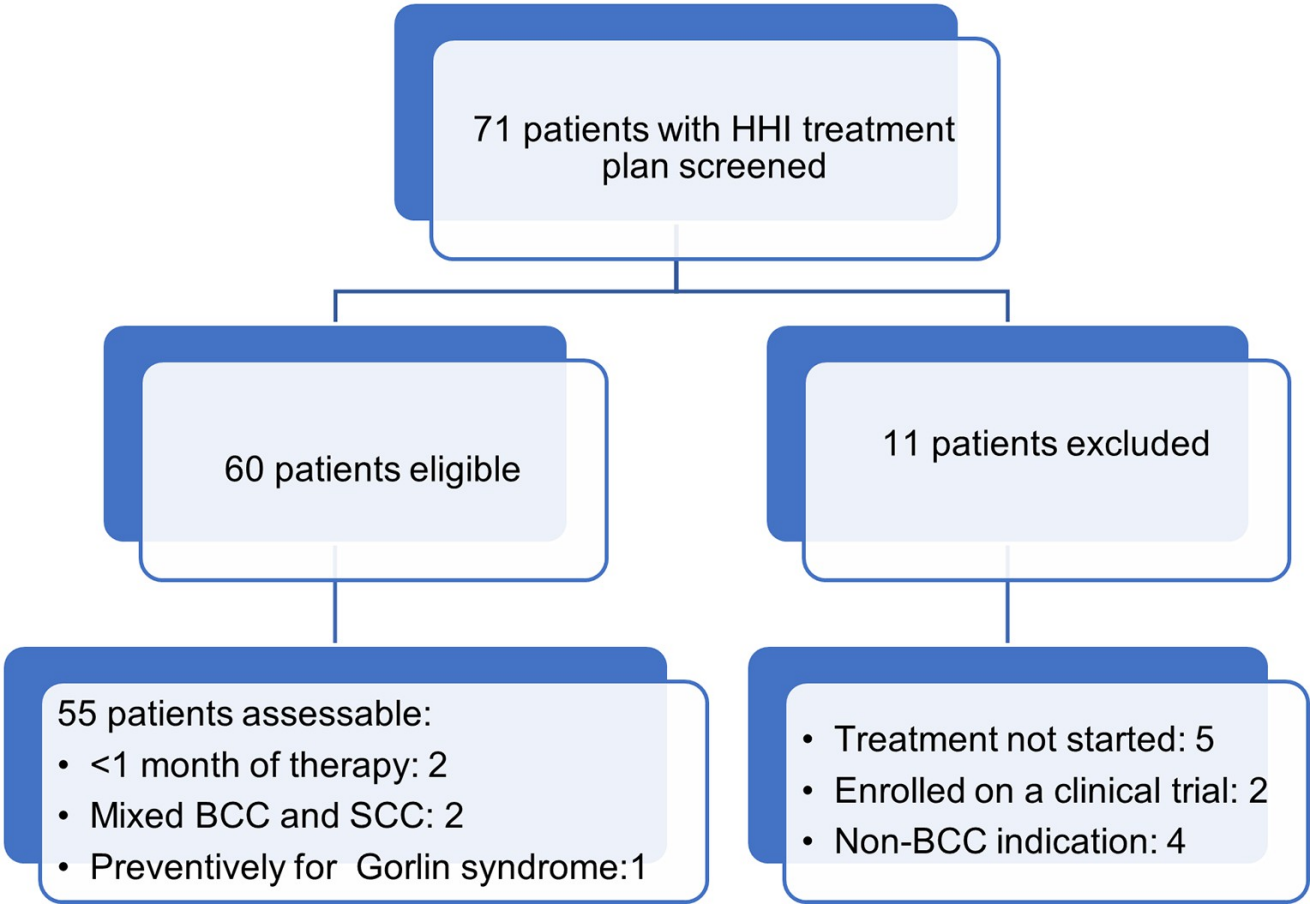
CONSORT diagram. Abbreviations: HHI, hedgehog inhibitor; BCC, basal cell carcinoma; SCC: squamous cell carcinoma.

Seventy-one patients were screened and 60 were included in this analysis (**Fig 1**). In all 60 cases, HHIs were started for a specific lesion in one of these categories: (1) inoperable or not suitable for radiation; (2) patient refused surgery or radiation; or (3) multiple BCCs arose with excessive frequency, making excision or Mohs surgery too frequent for tumor control. Patients’ characteristics are described in **Table 1**. Most patients were white males, and the median age was 65 years (range: 29–88 years). Three patients had BCNS (or Gorlin syndrome) [4] and one had Brooke-Spiegler syndrome [20]. Thirty-seven were treated with vismodegib and 23 with sonidegib.

**Table 1:** Patients baseline characteristics.

Characteristic	Vismodegib N = 37 (62%)	Sonidegib N = 23 (38%)	Total N = 60
**Median age, years (range)**	60 (29–86)	68 (49–88)	64 (29–88)
**Gender, n (%)**			
Male	24 (65)	12 (52)	36 (60)
**Race, n (%)**			
Asian	0	0	0 (0)
Black or African American	0	1 (4)	1 (1.7)
White	36 (97)	21 (92)	57 (95)
More than one race	0	1 (4)	1 (1.7)
Unknown	1 (3)	0	1 (1.6)
**Median laboratory values**	**Units (Range)**		
Creatinine clearance (ml/min)	94.6 (46–225.4)	90.9 (38.6–203.8)	87.7 (32.8–225.4)
Alkaline phosphatase (IU/L)	83 (39–777)	72 (41–184)	75 (37–777)
Alanine aminotransferase (IU/L)	17 (7–76)	14 (6–71)	16 (6–76)
Aspartate aminotransferase (IU/L)	18 (11–59)	16 (12–47)	18 (11–59)
Total bilirubin (mg/dL)	0.3 (0.5–1.3)	0.4 (0.2–1.3)	0.5 (0.2–1.3)
Creatine kinase (U/L)	88 (27–555)	71 (19–518)	88 (17–566)

### 3.2 Treatment

Thirty-seven patients (62%) received vismodegib and 23 (38%) received sonidegib (vismodegib was the first approved HHI for BCC). Initial treatment was at the discretion of the treating physician and was not systematically administered per prescription guidelines. While most patients started at the recommended dose, about 30% started with a lower dosage. Seventy percent of patients treated with vismodegib were initiated on the standard once-daily dosing, but 18% started vismodegib on a five-times-per-week frequency. Eighty-three percent of patients treated with sonidegib started the standard once-daily dosing, but 9% were prescribed a five-times-per-week frequency. **Table 2** shows the initial dose prescription for all members of this cohort. These numbers are too small to yield a statistically significant difference and represent the biases of the treating oncologists.

**Table 2:** Initial treatment regimen.

Initial Frequency	Vismodegib 150 mg (N = 37) n (%)	Sonidegib 200 mg (N = 23) n (%)
Once Daily	26 (70)	19 (83)
Five times a week	7 (18)	2 (9)
Other	4 (12)	2 (8)
Every Other Day	1 (3)	1 (4)
Three Times a Week	1 (3)	1 (4)
Twice a Week	1 (3)	ND
Dose Escalation^a^	1 (3)	ND

^a^ 4 times a week for 2 weeks, then increase to 5 times a week

ND, not done.

Dose reduction occurred in most patients (85%). Only 9 patients (15%) were able to maintain daily dosing. Nineteen patients (32%) were able to continue on HHIs for >6 months and entered the maintenance phase (**Table 3**). Seven patients crossed over to the alternate HHI due to various toxicities: 1 from sonidegib to vismodegib and 6 from vismodegib to sonidegib. Of these 7 patients, 3 remained on the crossover HHI for >6 months.

**Table 3:** Maintenance regimen and adverse event management.

*Maintenance regimen*	*Vismodegib (N = 7)*, *n (%)*	*Sonidegib (N = 12)*, *n (%)*	*Total* *(N = 19)*, *n (%)*
Once daily	4 (58)	5 (42)	9 (47)
Every other day	1 (14)	1 (8)	2 (11)
Six times a week	---	1 (8)	1 (5)
Twice weekly	1 (14)	---	1 (5)
Three times a week	1 (14)	4 (34)	5 (27)
Once weekly	---	1 (8)	1 (5)
***Proposed dose reductions for intolerance***			
**Drugs**	**Reduction Levels**	**Dosing**	**% Reduction**
Vismodegib/Sonidegib	First	5 times a week	30%
	Second	3 times a week	60%
	Third	1 time a week	85%
***Supplementation Recommendation***			
**Drugs**	**Supplementation**	**Strength**	**Daily Dose**
Calcium	Calcium carbonate	500 mg	500 mg
	Calcium citrate	250 mg	500 mg (2 tabs)
	Calcium gluconate	500 mg	1000 mg (2 tabs)
CoQ10		100 mg	100 mg

### 3.3 Outcome data

#### 3.3.1 Assessable patients

The cohort comprised 60 patients. Fifty-five patients were assessable for outcomes. Five patients were not assessable for the primary outcome for the following reasons: 2 patients did not take 1 month of therapy and stopped for subjective adverse events, 2 patients had a mixed BCC with squamous cell carcinoma (SCC) and died of SCC progression, and 1 patient was given HHI as secondary prevention in the setting of Gorlin syndrome. Of these 5 patients, 4 died as of the cutoff date and 1 remains alive (the patient with Gorlin syndrome).

#### 3.3.2 Progression-free survival

**Fig 2:**
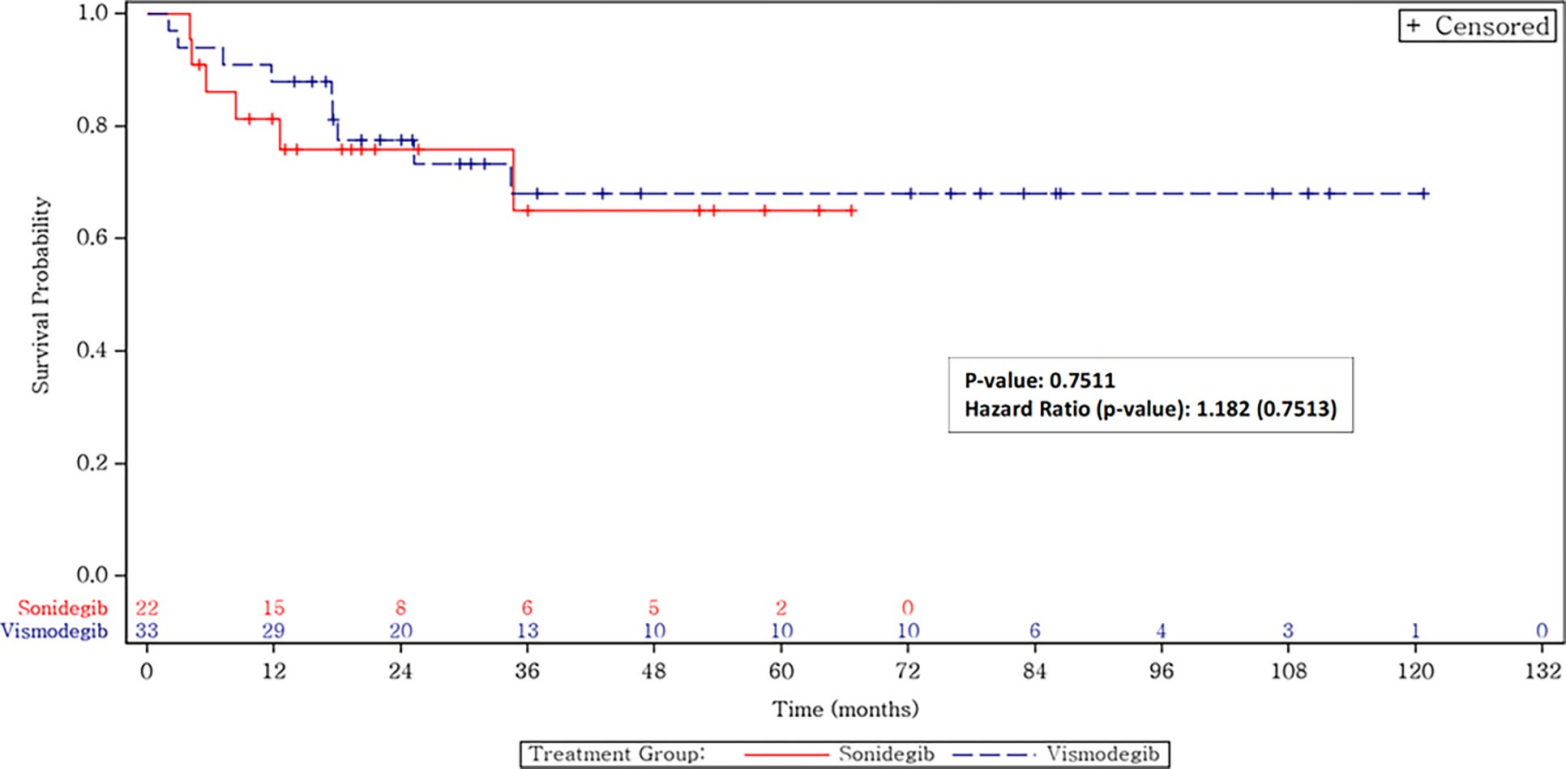
Progression-free survival by treatment.

For the whole cohort, the median PFS was not reached (95% CI, 34.7 months-not reached for sonidegib and 34.4 months-not reached for vismodegib) (**Fig 2**).

#### 3.3.3 Response rate

There were 25 (45.4%) complete responders and 9 (16.3%) partial responders, for an overall response rate (ORR) of 61.8%. Fourteen patients had stable disease (25.4%) and 7 (12.7%) had progression.

*Complete response*. Twenty-five patients saw a complete clinical response of the index BCC lesion. Of these, 19 (79%) remain in remission, 4 (17%) had new primary BCC leading to HHI discontinuation, 1 developed urothelial cancer (leading to death), and 1 developed metastatic disease treated further with cemiplimab with a complete response (CR).

*Partial response*. Nine patients had a partial remission with 2 patients (22.2%) having a progression on HHI therapy. Five patients received additional therapies (XRT and/or surgery) rendering them free of disease. Their PFS exceeds 39 months, which is very similar to patients obtaining a complete remission.

*Stable disease*. Fourteen patients had stable disease. Seven patients (50%) eventually progressed on HHI and received salvage immunotherapy, with 2 (28.5%) obtaining a complete response and 5 (71.5%) dying of BCC. One patient discontinued treatment and received topical therapies with continuing stable disease (SD). Two patients had surgery: 1 recurred after 9 months and the other remains in CR. Three patients died of unrelated causes. One patient with Gorlin syndrome switched from vismodegib to sonidegib and remains on treatment. Of these 14 patients, 2 are continuing on HHI with stable disease after 8 and 10 years, including the patient with Gorlin syndrome.

*Progression*. Seven patients did not respond to HHI and progressed. Two of these patients, who were treated initially with sonidegib, switched to cemiplimab and experienced a complete remission.

#### 3.3.4 Overall survival

**Fig 3:**
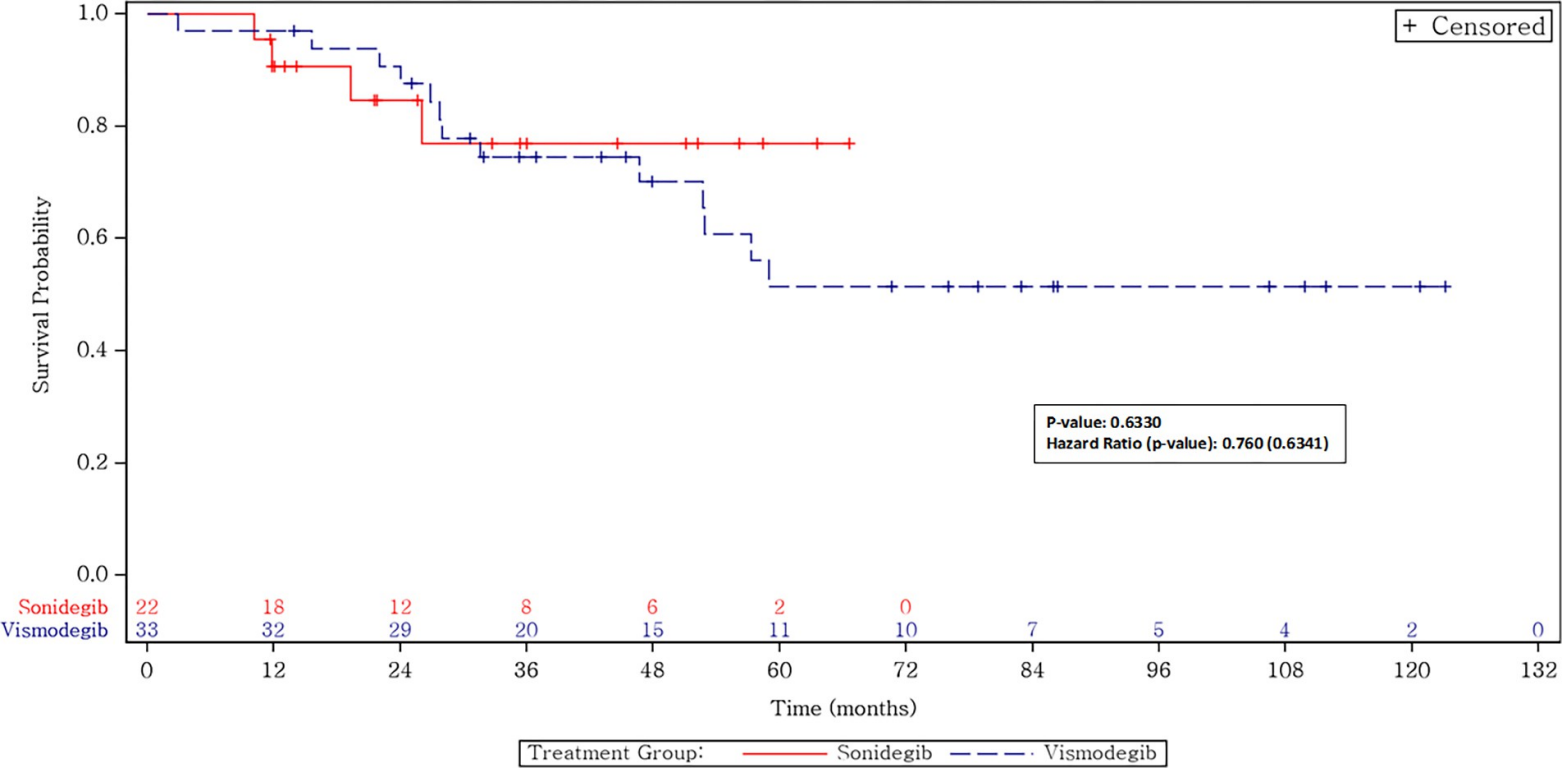
Overall survival.

**Figs 2 and 3** show the overall survival and the BCC-specific survival for patients treated with an HHI, broken out by the specific drug. The median survival was not reached for patients treated with either HHI. Of the 25 patients who obtained an initial CR, 3 patients died of non-BCC-related causes: 1 of COVID and 2 of other types of cancer. The BCC-specific survival rate for complete responders is thus 100%. Of the 9 patients who obtained a partial remission, 6 are alive and 3 died, 2 of different conditions and 1 of BCC progression after refusing further treatments. The BCC-specific survival rate in this group was thus 89%. Of the 14 patients with SD, 6 have died, 3 of BCC and 3 of other conditions. The BCC-specific survival rate was 78.6% for this group of patients who obtained SD. Of the 7 patients who progressed on HHI, 5 have died, 4 of BCC and 1 of an unrelated condition. Two patients were salvaged with cemiplimab and are alive. The BCC-specific overall survival rate for patients with progression on HHI was thus 42.8%.

#### 3.3.5 Recurrence

Of the 25 patients obtaining an initial CR, 7 had recurrences or new BCC later (28%). Of these 7 patients, 4 had a new primary BCC while on treatment, 1 had a new metastatic progression while still on HHI, and 2 recurred about 1 year after stopping vismodegib because of side effects. These two patients were rechallenged with sonidegib and achieved a CR afterward.

Of the 9 partial responders, 2 died of other causes and 1 of BCC. Of the remaining 6 patients, 2 were lost to follow-up and are not known to have had a recurrence, including a patient with Gorlin syndrome; 1 remains on treatment after more than 3 years; and 1 was treated with XRT and 1 with surgery, without recurrences. Only 1 patient who had metastatic disease when starting on HHI recurred after 12 months, and was then subsequently treated with cemiplimab and achieved a CR.

Upon progression, 14 patients received subsequent immunotherapy, 6 had salvage surgery, 3 underwent radiotherapy, and 2 received cetuximab with chemotherapy. Of the patients who received immunotherapy when they progressed, 9 patients (64%) received cemiplimab with a 56% CR and 36% received alternative immune checkpoint inhibitors; only 1 patient had a CR on the combination of ipilimumab and nivolumab (**S1 Table**). Three of 6 patients (50%) who had salvage surgeries did not experience recurrence.

### 3.4 Adverse events

Adverse events are shown in **S2 Table** and are as expected. The most common treatment-related adverse events with the initial treatment were myalgia/muscle spasm (46%), dysgeusia (37%), fatigue (30%), gastrointestinal effects (28%), weight loss (15%), and alopecia (15%). In the crossover group, the adverse event rates were similar with myalgia/muscle spasm (43%), fatigue (29%), and weight loss (28%) being the most common.

Approximately 63% of patients required at least one treatment interruption due to adverse events. Of the 24 patients who had treatment resumption upon toxicity resolution, 58% required dose reduction. A higher number of patients in the vismodegib group (59%) required changes in the regimen versus those in the sonidegib group (24%). In addition, the rate of treatment discontinuation was higher in the vismodegib group (30%) compared to the sonidegib group (9%). Of 60 patients, 7 patients (12%) were switched from one HHI to another due to intolerance (71%) or progression (29%).

Nineteen patients received calcium supplementation for either prevention (63%) or treatment (37%) of HHI-induced myalgia/muscle spasm. Of them, 8 patients received CoQ10 along with calcium supplementation. Patients who received prophylactic calcium and/or CoQ10 supplementation required fewer dose reductions (17%) than patients who did not receive prophylaxis (42%). However, the incidences of myalgia/muscle spasm did not differ between the prophylaxis and no-prophylaxis groups (42% and 46%, respectively).

Nineteen patients met our criteria for maintenance and were long-term HHI users. **Table 3** describes the dosing details for this population. More patients were receiving sonidegib (63%) compared to vismodegib (37%). Most of these patients tolerated the full dosing, but about half needed dose adjustments to remain on long-term HHI treatment.

### 3.5 Insurance status

About 93% of patients had insurance coverage for HHIs, but 7% did not. However, they were either eligible for manufacturer or non-manufacturer assistance, making HHIs accessible to all patients included in the study. It is important to note that even patients with HHI insurance coverage could also receive assistance, depending on the remaining amount not covered by insurance. Overall, 30% of patients received non-manufacturer assistance and 28% of patients received assistance from drug manufacturing companies. Forty-two percent of patients did not require any further assistance beyond insurance coverage (**S3 Table**).

## 4. Discussion

Vismodegib was the first selective small-molecule inhibitor approved in 2012 for laBCC or mBCC based on the ERIVANCE clinical trial results [21]. Thirty-three patients with mBCC and 71 with laBCC were enrolled and treated with vismodegib at 150 mg daily. ORR was 48.5% in the mBCC group (all partial responses) and 60.3% in the laBCC group, with 20 patients having a complete response and 18 a partial response. There were no differences in response per patient subgroups, including aggressive histologic subtypes such as infiltrative BCC. The median duration of response and OS were 14.8/26.2 and 33.4 months/not reached for mBCC/laBCC, respectively. Most common treatment-emergent AEs of any grade (**S2 Table**) included muscle spasms (71%), alopecia (69%), dysgeusia (58%), weight loss (54%), fatigue (45%), and nausea (34%). Grade ≥3 AEs occurred in 58 patients (56%) and were weight decrease (9%) and muscle spasms (6%). All other grade ≥3 AEs (fatigue, decreased appetite, diarrhea, and incidental SCC) occurred in <5% of patients. Thirteen of 104 patients (13%) had an adverse event leading to discontinuation of the study drug. None of the 33 observed deaths (31.7%) were related to vismodegib. Four other trials confirmed these results [22–25].

Sonidegib was the second HHI approved in 2015 for laBCC based on the results of the BOLT trial [26]. Out of 230 enrolled patients with laBCCs or mBCCs, 79 were randomized to 200 mg and 151 to 800 mg with ORRs of 56% and 46% for laBCC and 8% and 17% for mBCC, respectively. Median durations of response with 200 mg daily were 15.7 months for laBCC and 18.1 months for mBCC. Grade 3 toxicities were higher in the 800-mg compared to the 200-mg cohort with increased creatine kinase (CK) (10% vs. 3%), weight loss (6% vs. 5%), muscle spasms (5% each), decreased appetite (4% vs. 1%), nausea (3% vs. 1%), and fatigue (2% vs. 1%). The 200-mg dose was approved by the FDA for the treatment of laBCC in patients who are not candidates for surgery or radiation therapy.

**Fig 4:**
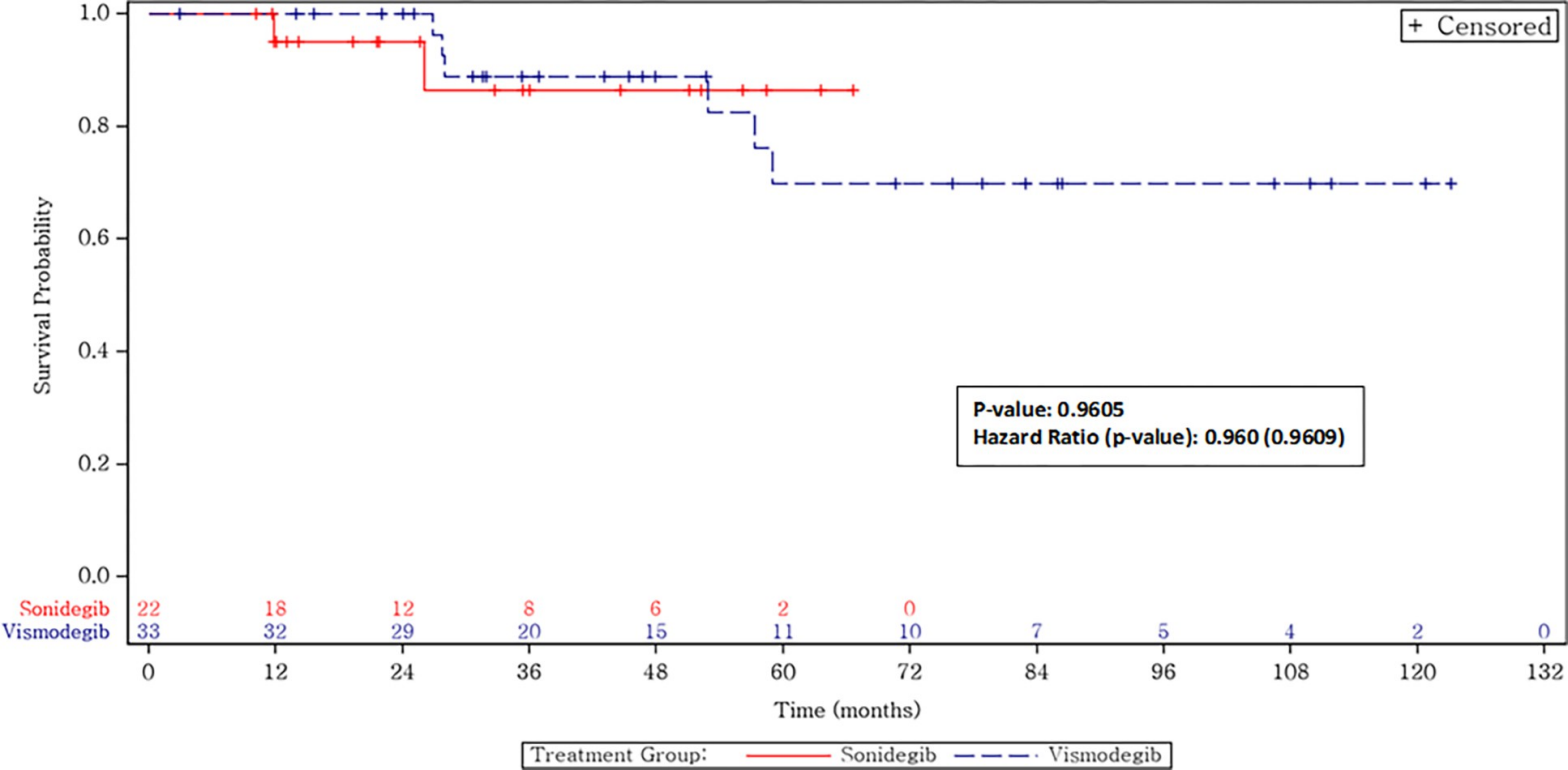
BCC-specific survival.

Our real-world data has similar outcomes, combining all stages of disease, with a 62% response rate and a progression-free survival that has not yet been reached with a 95% CI of about 34.5 months to not reached for both drugs. The major strength of our study is the focus on a real-world, post-marketing clinical experience in patients with various presentations of BCC, including patients with Gorlin syndrome, who were also responsive to HHIs [27]. Our cohort demonstrates confirmation of HHI activity in a typical oncology clinic population. There was no statistical difference found in efficacy between vismodegib and sonidegib, congruent with the results from prior landmark studies (**Figs 2–4**). In addition, similar to the BOLT study, the dosing modulation that was necessary for tolerability did not influence outcomes, as there was no significant difference when patients were treated with a lower initial regimen compared to once-daily dosing.

**S2 Table** also shows similar adverse events to those reported in the BOLT and ERIVANCE trials. However, our safety analysis showed significant differences in adverse events, with fewer dosing changes needed for sonidegib than for vismodegib (24% vs. 59%), and a rate of treatment discontinuation that was higher for vismodegib compared to sonidegib (30% vs. 9%). Our data strongly suggest that sonidegib is better tolerated than vismodegib and is equally efficacious. Further, 63% of patients receiving maintenance regimens were on sonidegib because sonidegib is better tolerated than vismodegib (**Table 3**).

There are no recommendations regarding the management of drug-related toxicities per the manufacturers’ labeling despite the high incidence of treatment-emergent AEs seen in the ERIVANCE and BOLT phase II trials [26, 28]. In our study, we show that calcium with or without CoQ10 supplementation decreased the number of dose reductions due to HHI-associated muscle spasms allowing patients to remain on therapy for longer periods of time.

Our study also informs about outcomes of patients who did not respond to HHIs and what salvage regimens were offered. Cemiplimab immunotherapy, radiotherapy, and surgery each have a role in refractory situations. In addition, the utility of neoadjuvant vismodegib was demonstrated in the phase II VISMONEO trial, which facilitated surgical downstaging in patients with locally advanced BCC [29]. Some BCCs are refractory to all treatment modalities. Longer exposure to HHIs might delay disease progression and prevents exhausting subsequent lines of therapy. Too many interruptions and reductions may lead to a decrease in median treatment duration and overall response rates. This association has been demonstrated with vismodegib in a study that assessed its safety and efficacy in a laBCC patient population [28, 30]. In our exploratory analyses, longer durations of treatment exposure were seen in patients with a reduced initial regimen without escalation and in patients with lower weekly dosing (3 or 5 times a week). Those on a daily regimen (7 days per week) required more interruptions and dose reductions.

While insurance approval was obtained for most patients (93%), at least one-third of the patients had a high copay (**S3 Table**). Two-thirds of the patients required non-manufacturer program assistance (usually foundation grants) or manufacturer assistance (Sun Pharmaceutical Industries for sonidegib or Genentech for vismodegib) to access HHI.

Our study has some limitations. It is a single-center retrospective review that was limited by missing data and inconsistent documentation in the EMR. The sample size was small. The treatment drug for the patients was picked based on the provider’s preference. Selecting an initial treatment regimen and decisions to interrupt treatment, adjust the regimen, or reduce the dose were not standardized. While a larger prospective trial should be conducted to truly assess the differences in efficacy and tolerability between the two hedgehog pathway inhibitors and to confirm our observations, it is not practical, given the low prevalence of such advanced BCC and the similarity of outcomes between the two drugs.

## 5. Conclusion

Overall, the real-world experience with HHIs in patients with laBCC or mBCC showed efficacy and safety results consistent with those seen in phase II trials. Our study suggests that sonidegib may be a better treatment option compared to vismodegib due to better tolerability with similar efficacy. The most common adverse events seen were muscle spasms, dysgeusia, weight loss, fatigue, GI effects, and alopecia. A less frequent dosing regimen may enhance tolerability and allow patients to stay on treatment longer while maintaining a steady response. In addition, calcium and/or CoQ10 supplementation may also help to decrease reductions due to HHI-associated myalgia/muscle spasm.

## Supporting information

S1 Table Secondary endpoints. (DOCX)

S2 Table Adverse events [28, 30, 31]. (DOCX)

S3 Table Insurance status. (DOCX)
