# Supplementary material for: Hedgehog pathway inhibitors for locally advanced and metastatic basal cell carcinoma: A real-world single-center retrospective review
Source: PLoS One. 2024 Apr 30;19(4):e0297531. doi: 10.1371/journal.pone.0297531 (PMC11060576; doi:10.1371/journal.pone.0297531)
Supplement: S1 Table — (DOCX) [file pone.0297531.s001.docx]

**Supplemental Table 1. Secondary Endpoints**

| **Subsequent Lines of Therapy** | **Patients, No. (%)** | **Response** |
| --- | --- | --- |
|  | N=25 (42%) |  |
| Second line  Cemiplimab  Pembrolizumab  Nivolumab  Ipilimumab/nivolumab  Cetuximab/Paclitaxel  Sorafenib  Surgery  XRT | 9 (36)  2 (8)  2 (8)  1 (4)  1 (4)  1 (4)  6 (24)  3 (12) | 5 CR (56%)  No  No  CR  SD  No  3 CR (50%)  1 CR (33%) |
| Next lines^a^  Second  Third | 4 patients  1  3 | No  No |

^a^ XRT, PD1 monoclonal but not cemiplimab, ipilimumab-based, and/or carboplatin/paclitaxel/cetuximab
