# Supplementary material for: Hedgehog pathway inhibitors for locally advanced and metastatic basal cell carcinoma: A real-world single-center retrospective review
Source: PLoS One. 2024 Apr 30;19(4):e0297531. doi: 10.1371/journal.pone.0297531 (PMC11060576; doi:10.1371/journal.pone.0297531)
Supplement: S2 Table — (DOCX) [file pone.0297531.s002.docx]

**Supplemental Table 2. Adverse Events**

| **Adverse event** | **OSUCCC Initial %**  **(N=60)** | **OSUCCC Crossover %**  **(N=7)** | **ERIVANCE %**  *vismodegib*^[28, 30]^  **(N=104)** | **BOLT %**  *sonidegib*^[31]^  **(N=230)** |
| --- | --- | --- | --- | --- |
| Muscle spasms/myalgia | 46 | 43 | 71 | 52 |
| Dysgeusia | 37 | --- | 58 | 41 |
| Fatigue | 30 | 29 | 45 | 29 |
| GI side effects | 28 | --- | Nausea 34  Diarrhea 28  Anorexia 29 | Nausea 35  Diarrhea 30  Anorexia 23 |
| Weight loss | 15 | 28 | 54 | 29 |
| Alopecia | 15 | --- | 69 | 49 |
| CK increase | 18 | ND | NR | 30^a^ |

^a^ Including grade 3/4 adverse event in 6.3% and 1.3% rhabdomyolysis not seen in our cohort

ND, Not done; NR: Not reported
