# Supplementary material for: Hedgehog pathway inhibitors for locally advanced and metastatic basal cell carcinoma: A real-world single-center retrospective review
Source: PLoS One. 2024 Apr 30;19(4):e0297531. doi: 10.1371/journal.pone.0297531 (PMC11060576; doi:10.1371/journal.pone.0297531)
Supplement: S3 Table — (DOCX) [file pone.0297531.s003.docx]

**Supplemental Table 3. Insurance Status**

|  | **Patients, No. (%)** |
| --- | --- |
| **Insurance Approval** | N=56 (93%) |
| **Copay**  $0  >$0 - $<100  >$100 - <$200  >$200 - <$500  >$500 - <$1000  >$1000  Unknown  **Non-manufacturer assistance**  **Manufacturer assistance** | 16 (29)  9 (16)  1 (2)  1 (2)  11 (19)  2 (4)  16 (29)  18 (30)  17 (28) |
